# Influence of School Year on Seasonality of Norovirus Outbreaks in Developed Countries

**DOI:** 10.1155/2017/9258140

**Published:** 2017-01-12

**Authors:** Roni Y. Kraut, Kate G. Snedeker, Oksana Babenko, Lance Honish

**Affiliations:** ^1^Department of Family Medicine, University of Alberta, Edmonton, AB, Canada T6G 2T4; ^2^Department of Public Health Sciences, University of Alberta, Edmonton, AB, Canada T6G 1C9; ^3^Surveillance and Reporting, Alberta Health Services, Edmonton, AB, Canada T2W 3N2; ^4^Environmental Public Health, Alberta Health Services, Edmonton, AB, Canada T5J 2Y2

## Abstract

Factors affecting the seasonal distribution of norovirus outbreaks are not well understood. This study examined whether grade school settings at the start of the school year may be a factor. We searched Ovid Medline from January 2002 to June 2014 for studies that provided all reported norovirus outbreaks in a developed country by month for a minimum of three years. Historical school years were obtained from verifiable sources. The start of the norovirus seasonal outbreak peak and peak outbreak month were determined for each study and compared to the start month of school. Northern hemisphere and southern hemisphere countries had a different norovirus seasonality and different school year structures (traditional compared to year round). In the two studies that provided outbreaks by age, outbreaks among children started several months before outbreaks in the adult population. The median number of months between school start and start of the seasonal outbreak peak was two months (interquartile range [IQR] = 2.0–3.0), while the median number of months between school start and peak outbreak month was four months (IQR = 3.0–4.0). These findings suggest the possibility the school setting at the start of the school year may be a factor in the seasonality of norovirus.

## 1. Introduction

Norovirus is a significant cause of gastroenteritis in developed countries worldwide [1]. In the United States alone it is estimated to cause 19–21 million cases of gastroenteritis, 400,000 emergency visits, and 570–800 deaths each year [2].

Norovirus cases in the northern hemisphere have been found to display a seasonal pattern, peaking in the fall and winter [3, 4]. A variety of factors have been studied to determine the etiology behind this seasonality, including environment (temperature, rainfall, watershed conditions, population density, and gross-domestic product) and agent (norovirus genotype); however, there is still no clear explanation [4–7].

The school setting at the start of the school year has not been well explored as a factor in the seasonality of norovirus. It may provide the right interplay of factors to potentiate outbreaks: a semiclosed environment, children in close quarters, and an influx of susceptible school children. This same combination of factors has already been found to propagate outbreaks on cruise ships [8, 9].

In addition, the school setting has already been found to be important in the transmission of influenza. For the 2009 pandemic, influenza cases decreased after summer vacation and increased after the resumption of the school year [10–12]. Further, studies across a diverse group of countries, including United States, Chile, Canada, and Israel, have demonstrated school closure is an effective means of decreasing influenza outbreaks among school age children [13, 14] and the entire population [10, 15].

The objective of this study was to explore whether the school setting may be a factor in the seasonality of norovirus outbreaks. Norovirus outbreaks in the province of Alberta, Canada, and selected nations were reviewed to determine if they temporally correlated with the start of the public school year. The preliminary findings were reported as a poster presentation at the 2014 Family Medicine Forum; this study elaborates on these findings [16].

## 2. Materials and Methods

### 2.1. Outbreak Data Sets

Ovid Medline was searched for papers published between January 1, 2002, and June 21, 2014, using subject headings and text words to retrieve articles related to the following concepts: norovirus, time of year, seasons, epidemiology, and disease outbreaks. The search strategy is provided in Supplementary Appendix 1 in Supplementary Material available online at https://doi.org/10.1155/2017/9258140. Case reports and results related to food safety, animals, and transplantation were excluded. Results were limited to the English language.

Studies were then retained if (1) they provided monthly norovirus outbreaks for a minimum of 3 years, (2) they were in a developed country [30], (3) they included all reported norovirus outbreaks from a specific region, laboratory, or reporting body, (4) they encompassed all norovirus strains, (5) outbreaks for each month were extractable, and (6) there was no age restriction on outbreak cases.

Each unique set of outbreaks was considered an outbreak set, with multicenter studies having the potential for multiple outbreak sets. From each outbreak set the following was extracted: setting of outbreaks, institution involved in collecting and reporting the outbreak data (surveillance institution), and monthly outbreak count. The total number of outbreaks extracted was compared to the total outbreaks listed in the study and was deemed to be complete if it was within ≤5% of the total outbreaks in the study.

The Alberta norovirus outbreaks from 2002–2012 were obtained from the Alberta Communicable Disease Reporting System. For each outbreak, date of first symptom onset, date the outbreak investigation opened, outbreak setting, and the reporting of Regional Health Authority were obtained.

The Health Panel of the University of Alberta Health Research Ethics Board approved this study.

### 2.2. Start Month of Seasonal Peak and Peak Outbreak Month

The calculations were adapted from methods described in Mounts et al. ([3] and Anthony Mounts, personal communication, 2014).

For each outbreak set, the monthly outbreak percentages were calculated by (1) computing the mean number of outbreaks for each month (the total number of outbreaks for a month, among all years, divided by the total number of years), (2) calculating the sum of the mean monthly outbreaks, and (3) dividing the mean outbreaks of each month by the sum of mean monthly outbreaks. The median outbreak percentage was calculated from the monthly outbreak percentages.

Next, for each outbreak set, the start month of the seasonal peak was determined, being the first month of at least three consecutive months where the monthly percentage of outbreaks was above the median. The peak outbreak month was also determined, being the month with the highest percentage of norovirus outbreaks.

### 2.3. Start Month of School

School year information, for all the years of each outbreak set, was obtained from a variety of sources. European school year information was taken from the yearly “Organization of School Time in Europe” reports [31]. New Zealand historical school year information was found on the New Zealand Ministry of Education Website [32]. For the United States, Google Trends™ was used to verify the start month of school, with the following search terms “back to school”, “school start”, “school year start”, and “start of school year”.

Surveys were sent to the Embassy of Japan in Canada (Japan), Hong Kong Education Bureau (Hong Kong), South Australia Department of Education and Child Development (Adelaide, Australia), Victoria Department of Education and Early Childhood Development (Victoria, Australia), and 22 of the school authorities in Alberta, which represented 70% of the total Alberta student population (Supplementary Appendix 2).

The median start date of school among all years was calculated for each outbreak set, except for Hong Kong, Japan, and Alberta; in these cases the start months were obtained directly from the survey. If the median start date was in the first half of the month, that month was considered the start month. If the median start date was in the second half of the month, the following month was considered the start month.

### 2.4. School Year Structure

Using the historical school year information, each of the data sets was determined to have either a “traditional school year” or “year round school year.” Traditional school year was defined as a school year with an extended vacation in the summer, a two-week winter vacation that included Christmas and New Year's day, and no other vacations in the school year greater than one week. Year round school year was defined as a school year divided into four terms, a two- to three-week vacation between each term, and approximately a six-week break at the end of the school year.

### 2.5. Statistical Analysis

For each outbreak set, the number of months between the median start month of school and the start month of the seasonal peak was calculated. With these results, the median number of months and interquartile range (IQR) among all data sets were calculated. In addition, for each outbreak set, the number of months between the start month of school and the peak outbreak month was calculated. With these results, the median number of months and interquartile range among all data sets were calculated.

Statistical methods, such as time series regression analysis and the spearman correlation coefficient, were considered for determining the significance of the relationship but not undertaken due to the minimal variation in the start month across the studies (the start month was the same in 15 of the 18 studies).

## 3. Results

### 3.1. Outbreak Sets

**Figure 1:**
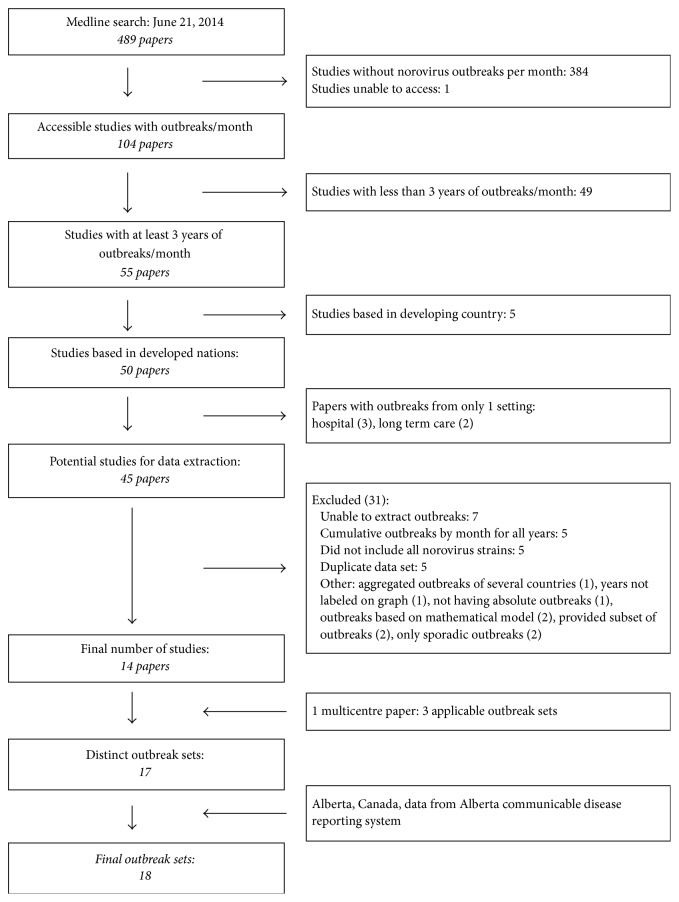
Outbreak set selection.

There were 479 studies from the Medline search, and 15 studies, with 17 unique outbreak sets, met the selection criteria. Including Alberta data, the total number of outbreak sets was 18 (Figure 1).  Table 1 shows the characteristics of each of the selected outbreak sets, including the percent of outbreaks in each setting.

### 3.2. Start Month of Seasonal Peak and Peak Outbreak Month

In the majority of outbreak sets, the start month of seasonal peak was in October/November (fall) and the peak outbreak month was approximately 2 months later in December/January (winter). However in Victoria, Australia, and New Zealand, the start month of seasonal peak was in June/July (winter) and the peak outbreak month was approximately 4 months later, in October (spring).

**Figure 2:**
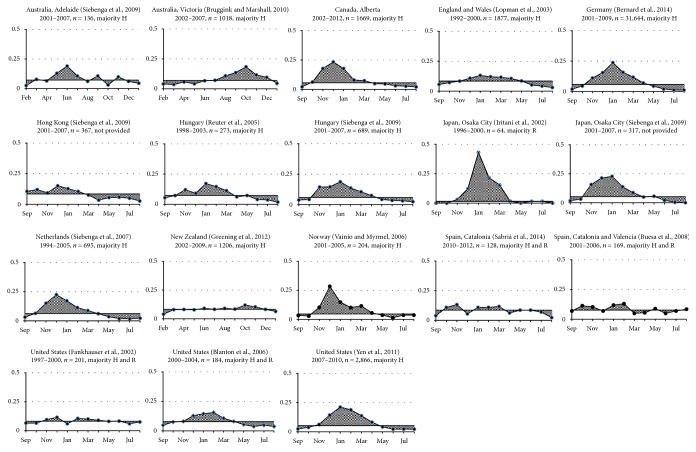
Monthly outbreak percentages of each outbreak set. Horizontal axis: month of the year, first month is school start month. Vertical axis: outbreak percentage. Solid vertical line: median outbreak percentage. H: hospital and long term care setting. R: restaurant and catered setting.

In addition, the majority of outbreak sets showed similar distribution of outbreaks, with the start month of seasonal peak preceding the peak outbreak month (Figure 2). In the United States (1997–2000) and Catalonia (2010–2012), however, the peak outbreak month preceded the start month of seasonal peak, and in Catalonia and Valencia (2001–2006), a start month of seasonal peak was not present. Supplementary Appendix 3 provides the details.

### 3.3. Start Month of School

The response rate to the Hong Kong, Japan, and Australia surveys was 100%. In Alberta, five school districts responded, representing 23.9% of the total school population in Alberta. In addition, a quarter of European school year dates were not obtained, due to several years without published Eurydice reports. The start month of school for all regions, except Australia and New Zealand, was determined to be September (Table 2). In Australia and New Zealand the start month of school was February.

### 3.4. School Year Structure

All of the regions, with the exception of Australia, New Zealand, and Germany, had a school year most consistent with a traditional school year. Australia and New Zealand outbreak sets had year round school years. Germany had characteristics of both traditional and year round school years.

### 3.5. Statistical Analysis

**Figure 3:**
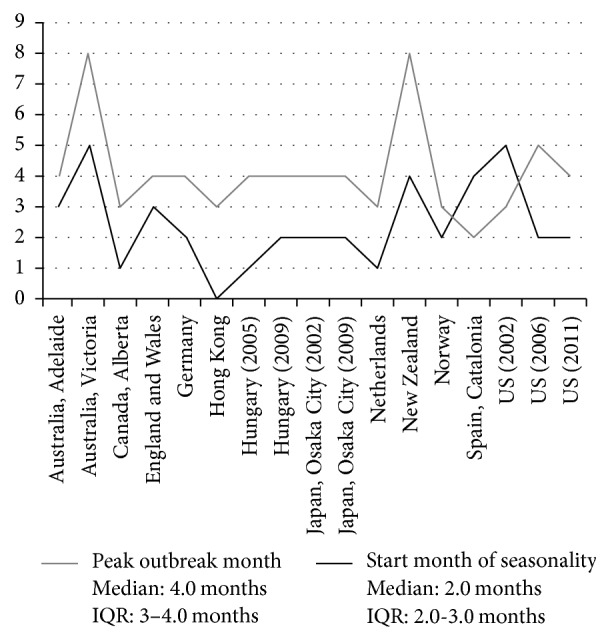
Start month of seasonal peak and peak outbreak month relative to start of school month by data set. Vertical axis: number of months after school year start month.

The median number of months between the start of school month and the start month of seasonal peak was two months (IQR 2.0-3.0) (Figure 3). The median number of months between the start of school month and the peak outbreak month was four months (IQR 3.0–4.0) (Figure 3).

## 4. Discussion

These findings, taken together, suggest the seasonality of norovirus induced gastroenteritis outbreaks may be related to the school setting at the start of the school year. Further, the outbreak sets used in the study provided credible outbreak data; all had three or more years of outbreak data and were collected by the regional or national surveillance institutions, and the majority did not have significantly skewed distributions.

The median time period between the start of the school year and the peak outbreak month, four months, corresponds to mathematical simulations by London and Yorke [33]. London and Yorke showed the delay of the start of mixing of new susceptibles to peak outbreaks in a population may be explained by the incubation period, the incubation period being the number of days from exposure to infectivity. Norovirus, with an incubation period of approximately two days [34], requires four months [33], whereas measles (incubation period of approximately 14 days [35]) requires approximately eight months [33].

The three outbreak sets without a clear seasonality, United States 2002 and Spain 2008 and 2014, had the majority of outbreak cases in settings other than health care institutions (Table 1). This appears consistent with previous research by Lopman et al. indicating that norovirus outbreaks in health institutions display a seasonal pattern, whereas the norovirus outbreaks in the community setting do not [18]. Alberta data showed similar results; only health institutions, schools, and child care settings displayed clear seasonality (Supplementary Appendix 4).

The German and Alberta outbreak sets were the only ones that provided information on seasonality of outbreaks by age group; the other studies primarily focused on genotype. Outbreaks in Germany started six weeks earlier in child care institutions and schools than in the general population [19]. Alberta data had similar findings; the start month of the seasonal peak of Alberta school outbreaks was two months earlier than the start month of outbreaks in health care settings (Supplementary Appendix 4).

The seasonality of norovirus cases in southern hemisphere countries is unclear. A recent systematic review and meta-analysis indicated there was not a consistent seasonality to outbreaks in the southern hemisphere, based on nine studies (four outbreaks and five sporadic cases) [4]. The authors suggested it may be due to a different epidemiologic pattern or lack of robust data, as only three studies had more than two years of data, and only one of these had population-wide data.

Our study included three southern hemisphere outbreak sets (Victoria Australia, New Zealand, and Adelaide Australia), with each having more than 3 years of population-wide monthly outbreaks. The first two clearly had a start month of seasonal peak in June/July and peak outbreak month in October, while Adelaide was different. However Adelaide had a total of 136 outbreaks over a seven-year time period, with 94 of these outbreaks in 2006. If the 2006 outbreak data is removed, the start month of seasonal peak is August and peak outbreak month is November (Supplementary Appendix 3).

**Figure 4:**
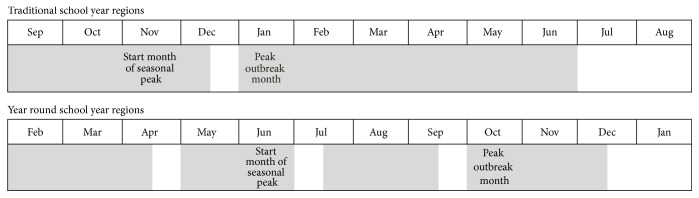
Comparison of type of school year with outbreak timing. School time is shaded and vacation time is shown in white. These dates are calculated based on median dates of the applicable data sets. If the majority of the median holiday dates was past mid-month, the holiday was considered to be the last 2 weeks of the month, if the majority of the median holiday dates was prior to mid-month, the holiday was considered to be in the first 2 weeks of the month.

The differing seasonality we found between the northern and southern hemisphere countries supports the previous 2013 systematic review, which found little association between weather conditions and seasonality of norovirus [4]. Alternatively, the school year may be contributing to these differences. Australia and New Zealand have year round schooling with a two-week holiday, two and a half months after the start of school. This may decrease the close contact and transmission of norovirus among school children. The northern hemisphere countries have a traditional school year, with a two-week holiday, three and a half months after the start of school (Figure 4). Further studies are required to both fully determine the seasonality of norovirus in the southern hemispheres and investigate the possible factors involved in the seasonality, including the school year.

This study is limited to analysis on a monthly basis instead of a weekly basis. Analysis on a weekly basis would better be able to determine the relationships between outbreaks and school year and allow for further statistical analysis. In addition, the data in this study was collected by more than 16 different surveillance organizations, each having their own individual surveillance methods. However, all the surveillance organizations are regional or national bodies, seasonality was generally consistent across the studies, and key studies in the past have examined outbreaks of different surveillance institutions together [1, 4].

The school setting at the start of the school year has the potential to be a factor in norovirus seasonality. The large German outbreak set with 31,644 outbreaks would seem ideal for future research, as there is substantial variation in school years across German states. In addition, further research could consider incidence, genotypes, and timing of norovirus cases in schools compared to health care institutions and whether improved norovirus surveillance and preventative measures in schools, including improved hand hygiene, lower the incidence of norovirus in the general population.

## Supplementary Material

Supplementary material provides detail on the literature search, questionnaires, outbreak set calculations, and Alberta outbreaks by setting.

## Figures and Tables

**Table 1:** Characteristics of the 18 outbreak sets.

Location	Surveillance institution	Total outbreaks	Time period	Median% of outbreaks/year (IQR)	Outbreak setting
Australia, Adelaide [1]	Institute of Medical and Veterinary Science	136	Jan 2001–Mar 2007	7% (4%–7%)	H primarily, other% not provided
Australia, Victoria [17]	Victoria Department of Human Services	1018	Jan 2002–Dec 2007	14% (9%–23%)	H 70%, other% not provided
Canada, Alberta	Alberta Health	1669	Jan 2002–Dec 2012	9% (8%-9%)	H 88%, R 3%, C 4%, P 1%, T 10%, O 4%
England and Wales [18]	Public Laboratory Service Communicable Disease Surveillance Centre	1877	Jan 1992–Dec 2000	11% (7%–16%)	H 79%, R 6%, C 4%, P 0%, T 8%, O 3%
Germany [19]	Robert Koch Institute, Department of Infectious Disease Epidemiology^a^	31644	Aug 2001–Jul 2009	6% (5%–18%)	H 60%, R <1%, C 10%, P 24%, T <1%, O 6%
Hong Kong [1]	Centre for Health Protection	367	Jan 2001–Mar 2007	11% (9%–15%)	Does not indicate
Hungary (2005) [20]	State Public Health Services and OEK National Centre for Epidemiology^a^	273	Jan 1998–Dec 2003	11% (3%–28%)	H 58%, R 3%, C 23%, P 0%, T 0%, O 16%
Hungary (2009) [1]	Regional Institute of State Public Health Services	689	Jan 2001–Mar 2007	12% (10%–17%)	H primarily, other% not provided
Japan, Osaka City (2002) [21]	Osaka City Institute of Public Health and Environmental Sciences^a^	64	Apr 1996–Mar 2000	20% (15%–29%)	H 0%, R 47%, C 0%, P 0%, T 24%, O 29%
Japan, Osaka City (2009) [1]	Osaka City Institute of Public Health and Environmental Sciences	317	Jan 2001–Mar 2007	20% (15%–29%)	Does not indicate
Netherlands [22]	National Institute of Public Health and the Environment	695	Dec 1994–Dec 2005	6% (4%–9%)	H 79%, R 8%, C 5%, P 0%, T 2%, O 6%
New Zealand [23]	New Zealand Ministry of Health	1206	Jan 2002–Dec 2009	14% (7%–16%)	H 64%, R 17%, C 5%, P 5%, T 4%, O 5%
Norway [24]	Norwegian Institute of Public Health	204	Jan 2001–Aug 2005	13% (12%–32%)	H 84%, other% not provided
Spain, Catalonia [25]	Public Health Agency of Catalonia	128	Jan 2010–Dec 2012	27% (25%–38%)	H 28%, R 30%, C 9%, P 9%, T 14%, O 10%
Spain, Catalonia and Valencia [26]	Not indicated	169	Jan 2001–Dec 2006	13% (12%–24%)	H 35%, R 16%, C 7%, P 10%, T 25%, O 7%
US (2002) [27]	Centre for Disease Control and Prevention	201	Jul 1997–Jun 2000	23% (18%–30%)	H 25%, R 39%, C 13%, P 0%, T 10%, O 12%
US (2006) [28]	Centre for Disease Control and Prevention	184	Jul 2000–Jun 2004	20% (19%–22%)	H 31%, R 28%, C 11%, P 2% T 18%, O 10%
US (2011) [29]	State and territorial public health departments	2866	Jan 2007–Apr 2010	27% (24%–28%)	H 65%, other% not provided

C, daycare, school, and camps; H, hospital and long term care institution; O, other; P, private home; R, restaurant and catering; T, travel hotel.

^a^Surveillance institution determined by author affiliation.

**Table 2:** School year characteristics of outbreak set regions.

Location	Source	School year type; holidays > 1 week	School year	
			Median start date (IQR)	Start month
Australia, South Australia	South Australia Department of Education and Child Development (survey)	Y	Jan 29 (28-29)	February
Australia, Victoria	Victoria Department of Education and Early Childhood Development (survey)	Y	Jan 29 (29-30)	February
Canada, Alberta	Alberta school authorities (survey)	T: CV, SuV	NA	September
England and Wales	Eurydice	T: CV, SV, SuV	Sep 4 (1–8)	September
Germany	Eurydice	U: AV, CV, SV, SuV	Aug 23 (16–25)	September
Hong Kong	Hong Kong Education Bureau (survey)	T: no data	NA	September
Hungary	Eurydice	T: CV, SuV	Sep 1 (Aug 31-Sep 1)	September
Japan^a^	Embassy of Japan in Canada (survey)	T: CV, SV, SuV	NA	September
Netherlands	Eurydice	T: CV, SuV	Aug 23 (22–25) p Aug 27 (26–29) s	September
New Zealand	New Zealand Ministry of Education website	Y	Feb 1 (Jan 29–Feb 7)	February
Norway	Eurydice	T: CV, SuV	Aug 19 (18-19)	September
Spain, Catalonia	Eurydice	T: CV, SuV	Sep 7 (7-7)	September
Spain, Catalonia and Valencia	Eurydice	T: CV, SuV	Sep 11 (9–15) p Sep 15 (15–17) s	September
US	Google trends	T: no data	NA	September

School year type: Y: year round, T: traditional, U: unclear.

Holidays: AV: autumn, CV: Christmas/New Years, SV: spring, SuV: summer.

Age level: p: primary, s: secondary.

^a^The start month of school is April in Japan; however September was used as this follows the longest school vacation.
