# Supplementary material for: Influence of School Year on Seasonality of Norovirus Outbreaks in Developed Countries
Source: Can J Infect Dis Med Microbiol. 2017 Jan 12;2017:9258140. doi: 10.1155/2017/9258140 (PMC5266842; doi:10.1155/2017/9258140)
Supplement: Supplementary file 1 — Supplementary material provides detail on the literature search, questionnaires, outbreak set calculations, and Alberta outbreaks by setting. [file 9258140.f1.docx]

**Appendix 1: Ovid medline search strategy**

1 incidence/
2 seasons/
3 (epidemiolog* or incidence or season* or month* or week*).ti.
4 1 or 2 or 3
5 exp Norovirus/
6 Caliciviridae Infections/
7 (norovirus* or norwalk or small round structured virus* or SRSV).ti.
8 5 or 6 or 7
9 4 and 8
10 ep.fs.
11 *disease outbreaks/
12 *Norovirus/
13 exp *Food Safety/
14 transplantation.ti. or exp Transplantation/
15 Animals/
16 10 and 11 and 12
17 9 or 16
18 13 or 14 or 15
19 17 not 18
20 limit 19 to (case reports or editorial or historical article or letter or news)
21 19 not 20
22 limit 21 to english language
23 limit 22 to yr="2002 -Current"

**Appendix 2: Questionnaires**

**Questionnaire: timing of school year in Osaka City, Japan**

Please answer the following questions regarding the school year in your school division during the years 1996 to 2000.

1.  It is our understanding that, for most elementary, junior high, and high schools, the school year in Japan begins on April 1 and is divided into three terms: April to July, September to December, and January to March.

Does this school year also apply to Osaka City schools during the years 1996 - 2000?

Please indicate “Yes” or “No” below.

If no, please explain.

2.  It is our understanding that, for most elementary, junior high, and high schools in Japan, there are 3 vacations each year that are longer that one week duration.  These are summer, winter and spring vacation.

Does this also apply to Osaka City schools during the years 1996 - 2000?

Please indicate “Yes” or “No” below.

If no, please explain.

3.  Please indicate the approximate dates of the summer, winter and spring vacations.

4.  It is our understanding that a large proportion of elementary, junior high, and high schools students go to school full time over the summer break.

Does this also apply to Osaka City during the years 1996 - 2000?

Please indicate “Yes” or “No” below.

If no, please explain.

5.  On average, approximately what percentage of kindergarten, elementary, junior high and high school students in your school division go to school full time over the summer break?

x

**Questionnaire: timing of school year in Alberta School Divisions**

Please answer the following questions regarding the school year in your school division during the years 2002 to 2012.

1.  For the years 2002 - 2012,  please indicate if the majority of students in your school district had a 'traditional' school year or a 'year round’ school year, **by placing an “X” next to the appropriate response.**

***Note:****‘Traditional’ school year:  a school year that ends with a 2-month summer holiday.*

*‘Year round’ school year:  a school year with a shorter summer holiday and multiple two-week vacations (e.g. during Fall, the Festive Season and at Spring Break)*

_____   The majority of the students in the school division had a ‘traditional’ school year during 2002-2012 **(proceed to question 2)**

_____   The majority of the students in the school division had a ‘year round’ school year during 2002-2012 **(skip to question 3)**

 _____  The type of school year changed during 2002 - 2012.  For part of this time period the majority of students had a 'year round' school year and for the other part the majority of students had a 'traditional' school year. **(complete question 2 and 3 for the years that each type of school year applied to the majority of students)**

_____   The majority of students had a school year different from both 'year round' and 'traditional' schooling during 2002-2012.  Please provide details below of the date the school year starts, the date the school year ends and the date and length of all vacations during the school year during 2002-2012:

Questions 2.a. – 2.j. are for school divisions in which the majority of students had a **traditional school year** during 2002-2012:

2.a.  For the majority of students in your school division, during the period 2002 - 2012, did the school year begin between the last week in August and the first week in September? Please answer 'yes' or 'no'.

2.b. If 'no', please provide the approximate school year start dates for the majority of students for each year between 2002 - 2012.

2.c. For the majority of students of schools in your school division, during the period 2002 - 2012, did summer vacation start between the last week in June and the first week in July? Please answer 'yes' or 'no'.

2.d If 'no' please provide the approximate start dates of summer vacation for the majority of students for each year between 2002 - 2012.

2.e. For the majority of students of schools in your school division, during the period 2002 – 2012, was winter vacation approximately 2 weeks in length between middle of December and early January?

2.f If 'no' please provide the approximate dates of winter vacation for the majority of students for each year between 2002 - 2012.

2.g. For the majority of students of schools in your school division, during the period 2002 – 2012, was spring vacation approximately 1 week in length between the months of March and April?  Please answer ‘yes’ or ‘no’.

2.h. If 'no' please provide the approximate dates of spring vacation for the majority of students for each year between 2002 - 2012.

2.i. This survey has asked about the timing of summer, winter and spring vacations in your school division.  Besides these vacations, were there other vacations greater than one week in duration occurring yearly for the majority of students, during the period 2002 - 2012?  Please answer 'yes' or 'no'.

2.j.  If ‘yes’ please provide the approximate date(s) of these vacations for the majority of students for each year between 2002 – 2012.

Questions 3.a. – 3.e. are for school divisions in which the majority of students had a **‘year round’ school year** during 2002-2012:

3.a.         For the majority of students, on what approximate date did the school year begin during 2002-2012?

3.b.       For the majority of students, on what approximate date did Fall vacation begin during 2002-2012, and how long was Fall vacation?

3.c.        For the majority of students, on what approximate date did Winter vacation begin during 2002-2012, and how long was Winter vacation?

3.d.        For the majority of students, on what approximate date did Spring vacation begin during 2002-2012, and how long was Spring vacation?

3.e.    For the majority of students, on what approximate date did Summer vacation begin during 2002-2012?

4.  Please provide any additional comments you may have about school year dates in your school division during 2002-2012 that pertain to the majority of students

**Appendix 3: Outbreaks of each outbreak set by month**

SMSP: start month of seasonal peak, POM: peak outbreak month

| **Australia, Adelaide** | Median monthly outbreak percentage: 0.072 | | | | | | |  | | | |  | |  | |  | |
| --- | --- | --- | --- | --- | --- | --- | --- | --- | --- | --- | --- | --- | --- | --- | --- | --- | --- |
| Month | 2001 | 2002 | 2003 | | 2004 | 2005 | 2006 | 2007 | | Avg/month | | | %/month | | | |  |
| February | 0 | 0 | 0 | | 0 | 0 | 3 | 1 | | 0.6 | | | 0.03 | | | |  |
| March | 0 | 0 | 0 | | 1 | 0 | 10 | 1 | | 1.7 | | | 0.08 | | | |  |
| April | 0 | 0 | 0 | | 0 | 0 | 9 | 1 | | 1.4 | | | 0.06529 | | | |  |
| May | 0 | 0 | 0 | | 1 | 2 | 14 | no data | | 2.8 | | | **0.13 SMSP** | | | |  |
| June | 0 | 0 | 1 | | 0 | 0 | 24 | no data | | 4.2 | | | **0.19 POM** | | | |  |
| July | 0 | 0 | 0 | | 0 | 0 | 14 | no data | | 2.3 | | | 0.11 | | | |  |
| August | 1 | 2 | 0 | | 0 | 0 | 5 | no data | | 1.3 | | | 0.06 | | | |  |
| September | 0 | 5 | 0 | | 1 | 1 | 7 | no data | | 2.3 | | | 0.11 | | | |  |
| October | 0 | 1 | 0 | | 1 | 2 | 0 | no data | | 0.7 | | | 0.03 | | | |  |
| November | 2 | 0 | 3 | | 5 | 2 | 1 | no data | | 2.2 | | | 0.10 | | | |  |
| December | 0 | 1 | 3 | | 1 | 2 | 1 | no data | | 1.3 | | | 0.06 | | | |  |
| January | 0 | 0 | 0 | | 0 | 0 | 6 | 1 | | 1 | | | 0.05 | | | |  |
|  | 3 | 9 | 7 | | 10 | 9 | 94 | 4 | | 22 | | | 1 | | | |  |
|  | Total outbreaks 136 | | | | |  |  |  | |  | | |  | | | |  |
| **Australia, Adelaide (2006 excluded)** | | | | Median monthly outbreak percentage: 0.057 | | | | | | | | |  |  |  |  |  |
| Month | 2001 | 2002 | 2003 | | 2004 | 2005 | 2007 | | Avg/month | | %/month | | | |  |  |  |
| February | 0 | 0 | 0 | | 0 | 0 | 1 | | 0.2 | | 0.02 | | | |  |  |  |
| March | 0 | 0 | 0 | | 1 | 0 | 1 | | 0.3 | | 0.04 | | | |  |  |  |
| April | 0 | 0 | 0 | | 0 | 0 | 1 | | 0.2 | | 0.02 | | | |  |  |  |
| May | 0 | 0 | 0 | | 1 | 2 | no data | | 0.6 | | 0.07 | | | |  |  |  |
| June | 0 | 0 | 1 | | 0 | 0 | no data | | 0.2 | | 0.02 | | | |  |  |  |
| July | 0 | 0 | 0 | | 0 | 0 | no data | | 0.0 | | 0.00 | | | |  |  |  |
| August | 1 | 2 | 0 | | 0 | 0 | no data | | 0.6 | | **0.07 SMSP** | | | |  |  |  |
| September | 0 | 5 | 0 | | 1 | 1 | no data | | 1.4 | | 0.17 | | | |  |  |  |
| October | 0 | 1 | 0 | | 1 | 2 | no data | | 0.8 | | 0.10 | | | |  |  |  |
| November | 2 | 0 | 3 | | 5 | 2 | no data | | 2.4 | | **0.29 POM** | | | |  |  |  |
| December | 0 | 1 | 3 | | 1 | 2 | no data | | 1.4 | | 0.17 | | | |  |  |  |
| January | 0 | 0 | 0 | | 0 | 0 | 1 | | 0.17 | | 0.02 | | | |  |  |  |
|  | 3 | 9 | 7 | | 10 | 9 | 4 | | 8 | | 1 | | | |  |  |  |
|  | Total outbreaks 42 | | | |  |  |  | |  | |  | | | |  |  |  |

| **Australia, Victoria** | | Median monthly outbreak percentage: 0.0694 | | | | | |  |
| --- | --- | --- | --- | --- | --- | --- | --- | --- |
| Month | 2002 | 2003 | 2004 | 2005 | 2006 | 2007 | Total | %/month |
| February | 5 | 5 | 6 | 5 | 12 | 8 | 41 | 0.04 |
| March | 5 | 1 | 7 | 6 | 16 | 1 | 36 | 0.04 |
| April | 6 | 1 | 11 | 6 | 28 | 6 | 58 | 0.06 |
| May | 3 | 0 | 8 | 1 | 23 | 7 | 42 | 0.04 |
| June | 3 | 0 | 8 | 4 | 50 | 3 | 68 | 0.0675 |
| July | 5 | 2 | 13 | 6 | 34 | 12 | 72 | **0.0714 SMSP** |
| August | 7 | 8 | 25 | 7 | 19 | 43 | 109 | 0.11 |
| September | 16 | 7 | 29 | 5 | 16 | 64 | 137 | 0.14 |
| October | 35 | 9 | 16 | 9 | 44 | 72 | 185 | **0.18 POM** |
| November | 31 | 10 | 19 | 6 | 37 | 14 | 117 | 0.12 |
| December | 13 | 15 | 8 | 25 | 24 | 12 | 97 | 0.10 |
| January | 1 | 3 | 5 | 4 | 16 | 17 | 46 | 0.05 |
|  | 130 | 61 | 155 | 84 | 319 | 259 | 1008 | 1 |

| **Canada, Alberta** | | Median monthly outbreak percentage: 0.0557 | | | | | | |  |  |  |  |  |
| --- | --- | --- | --- | --- | --- | --- | --- | --- | --- | --- | --- | --- | --- |
| Month | 2002 | 2003 | 2004 | 2005 | 2006 | 2007 | 2008 | 2009 | 2010 | 2011 | 2012 | Total | %/month |
| September | 1 | 0 | 1 | 1 | 12 | 5 | 3 | 3 | 3 | 5 | 3 | 37 | 0.02 |
| October | 14 | 0 | 11 | 5 | 28 | 4 | 12 | 2 | 7 | 9 | 13 | 105 | **0.0629 SMSP** |
| November | 45 | 10 | 39 | 10 | 87 | 9 | 27 | 3 | 17 | 28 | 21 | 296 | 0.18 |
| December | 53 | 15 | 56 | 6 | 60 | 20 | 38 | 10 | 48 | 33 | 49 | 388 | **0.23 POM** |
| January | 3 | 37 | 18 | 49 | 11 | 35 | 20 | 37 | 17 | 31 | 38 | 296 | 0.18 |
| February | 1 | 11 | 8 | 14 | 7 | 20 | 9 | 23 | 13 | 18 | 10 | 134 | 0.08 |
| March | 3 | 5 | 7 | 18 | 12 | 20 | 10 | 17 | 7 | 13 | 12 | 124 | 0.07 |
| April | 1 | 3 | 2 | 6 | 14 | 10 | 13 | 9 | 4 | 13 | 6 | 81 | 0.05 |
| May | 0 | 1 | 2 | 3 | 22 | 10 | 9 | 2 | 9 | 11 | 8 | 77 | 0.05 |
| June | 4 | 3 | 2 | 4 | 11 | 6 | 3 | 6 | 7 | 3 | 2 | 51 | 0.03 |
| July | 7 | 3 | 1 | 4 | 10 | 3 | 3 | 8 | 4 | 1 | 1 | 45 | 0.03 |
| August | 4 | 0 | 1 | 1 | 16 | 1 | 3 | 7 | 0 | 1 | 1 | 35 | 0.02 |
|  | 136 | 88 | 148 | 121 | 290 | 143 | 150 | 127 | 136 | 166 | 164 | 1669 | 1 |

| **England & Wales** | | | Median monthly outbreak percentage: 0.08428 | | | | | | |  | |  | |  |  | |  |
| --- | --- | --- | --- | --- | --- | --- | --- | --- | --- | --- | --- | --- | --- | --- | --- | --- | --- |
| Month | 1992 | 1993 | | 1994 | 1995 | 1996 | 1997 | 1998 | 1999 | | 2000 | | Total | | | %/month |  |
| September | 9 | 9 | | 14 | 13 | 7 | 13 | 12 | 14 | | 13 | | 105 | | | 0.06 |  |
| October | 9 | 18 | | 21 | 11 | 21 | 11 | 16 | 15 | | 11 | | 134 | | | 0.07 |  |
| November | 13 | 8 | | 13 | 11 | 14 | 20 | 39 | 31 | | 8 | | 157 | | | 0.0836 |  |
| December | 6 | 11 | | 12 | 34 | 28 | 13 | 43 | 36 | | 20 | | 203 | | | **0.11 SMSP** |  |
| January | 1 | 12 | | 13 | 57 | 41 | 10 | 25 | 39 | | 47 | | 245 | | | **0.13 POM** |  |
| February | 2 | 11 | | 13 | 29 | 39 | 25 | 18 | 29 | | 59 | | 225 | | | 0.12 |  |
| March | 0 | 12 | | 17 | 47 | 62 | 7 | 11 | 25 | | 37 | | 217 | | | 0.12 |  |
| April | 2 | 15 | | 7 | 51 | 42 | 13 | 15 | 19 | | 34 | | 198 | | | 0.11 |  |
| May | 0 | 7 | | 13 | 49 | 27 | 9 | 9 | 17 | | 28 | | 159 | | | 0.08 |  |
| June | 4 | 4 | | 10 | 40 | 9 | 5 | 8 | 5 | | 12 | | 97 | | | 0.05 |  |
| July | 5 | 11 | | 9 | 9 | 7 | 7 | 10 | 6 | | 13 | | 78 | | | 0.04 |  |
| August | 1 | 9 | | 8 | 7 | 9 | 3 | 8 | 4 | | 9 | | 57 | | | 0.03 |  |
|  | 53 | 128 | | 149 | 358 | 307 | 136 | 214 | 239 | | 291 | | 1875 | | | 1 |  |

| **Germany** | | | | Median monthly outbreak percentage: 0.06 | | | | | | | | | | | | | | | | | | | | |  | | |  | | | | | |  | | |  | | | | | | | |  | | |
| --- | --- | --- | --- | --- | --- | --- | --- | --- | --- | --- | --- | --- | --- | --- | --- | --- | --- | --- | --- | --- | --- | --- | --- | --- | --- | --- | --- | --- | --- | --- | --- | --- | --- | --- | --- | --- | --- | --- | --- | --- | --- | --- | --- | --- | --- | --- | --- |
| Month | 2001 | | | | | | 2002 | | 2003 | | 2004 | | | 2005 | | | | 2006 | | | | | 2007 | | | | | | 2008 | | | 2009 | | | | | | Avg/month | | | | %/month | | | |  |  |
| September | 17 | | | | | | 34 | | 34 | | 86 | | | 52 | | | | 52 | | | | | 138 | | | | | | 190 | | | no data | | | | | | 75 | | | | 0.02 | | | |  |  |
| October | 17 | | | | | | 241 | | 17 | | 172 | | | 34 | | | | 172 | | | | | 431 | | | | | | 328 | | | no data | | | | | | 177 | | | | 0.05 | | | |  |  |
| November | 17 | | | | | | 466 | | 86 | | 431 | | | 103 | | | | 345 | | | | | 1,293 | | | | | | 707 | | | no data | | | | | | 431 | | | | **0.11 SMSP** | | | |  |  |
| December | 34 | | | | | | 345 | | 69 | | 534 | | | 121 | | | | 690 | | | | | 1,793 | | | | | | 1,241 | | | no data | | | | | | 603 | | | | 0.15 | | | |  |  |
| January | no data | | | | | | 138 | | 431 | | 121 | | | 603 | | | | 310 | | | | | 1,207 | | | | | | 2,603 | | | 1,983 | | | | | | 925 | | | | **0.24 POM** | | | |  |  |
| February | no data | | | | | | 86 | | 224 | | 155 | | | 310 | | | | 362 | | | | | 1,034 | | | | | | 1,310 | | | 1,431 | | | | | | 614 | | | | 0.16 | | | |  |  |
| March | no data | | | | | | 69 | | 172 | | 172 | | | 224 | | | | 310 | | | | | 655 | | | | | | 879 | | | 1,172 | | | | | | 457 | | | | 0.12 | | | |  |  |
| April | no data | | | | | | 86 | | 86 | | 69 | | | 103 | | | | 172 | | | | | 603 | | | | | | 638 | | | 431 | | | | | | 274 | | | | 0.07 | | | |  |  |
| May | no data | | | | | | 52 | | 34 | | 52 | | | 86 | | | | 138 | | | | | 431 | | | | | | 276 | | | 293 | | | | | | 170 | | | | 0.04 | | | |  |  |
| June | no data | | | | | | 34 | | 17 | | 34 | | | 52 | | | | 86 | | | | | 138 | | | | | | 138 | | | 138 | | | | | | 80 | | | | 0.02 | | | |  |  |
| July | no data | | | | | | 34 | | 17 | | 34 | | | 34 | | | | 34 | | | | | 86 | | | | | | 86 | | | 52 | | | | | | 47 | | | | 0.0122 | | | |  |  |
| August | 9 | | | | | | 17 | | 17 | | 52 | | | 34 | | | | 52 | | | | | 103 | | | | | | 69 | | | no data | | | | | | 44 | | | | 0.0113 | | | |  |  |
|  | 95 | | | | | | 1,603 | | 1,207 | | 1,914 | | | 1,759 | | | | 2,724 | | | | | 7,914 | | | | | | 8,466 | | | 5,500 | | | | | | 3,898 | | | | 1 | | | |  |  |
|  | Total outbreaks 31,181 | | | | | | | | | |  | | | | |  | | |  | | | | | | | | | | | | | |  | | | | | |  | | | |  | | | |  |
|  |  | | | | |  | | |  |  | | |  | | | |  | | | |  | | | | |  | | | |  | | | | | |  | | | | |  | | | | |  |  |
| **Hong Kong** | | | Median monthly outbreak percentage: 0.08 | | | | | | | | | | | | | | | | | | |  | | | | |  | | | | | | | |  | | | | |  |  |  |  |  |  |  |  |
| Month | | 2001 | | | 2002 | | | 2003 | | | | 2004 | | | 2005 | | | | | 2006 | | | | 2007 | | | | | | | Avg/month | | | | | | | %/month | | | | | |  |  |  |  |
| September | | 5 | | | 3 | | | 0 | | | | 22 | | | 2 | | | | | 5 | | | | no data | | | | | | | 6.1 | | | | | | | **0.11 SMSP** | | | | | |  |  |  |  |
| October | | 17 | | | 11 | | | 1 | | | | 10 | | | 0 | | | | | 3 | | | | no data | | | | | | | 7.0 | | | | | | | 0.12 | | | | | |  |  |  |  |
| November | | 2 | | | 16 | | | 3 | | | | 5 | | | 2 | | | | | 5 | | | | no data | | | | | | | 5.4 | | | | | | | 0.09 | | | | | |  |  |  |  |
| December | | 4 | | | 3 | | | 14 | | | | 1 | | | 17 | | | | | 13 | | | | no data | | | | | | | 8.8 | | | | | | | **0.15 POM** | | | | | |  |  |  |  |
| January | | 0 | | | 8 | | | 6 | | | | 6 | | | 7 | | | | | 5 | | | | 19 | | | | | | | 7.4 | | | | | | | 0.13 | | | | | |  |  |  |  |
| February | | 0 | | | 4 | | | 9 | | | | 7 | | | 5 | | | | | 10 | | | | 8 | | | | | | | 6.0 | | | | | | | 0.10 | | | | | |  |  |  |  |
| March | | 0 | | | 3 | | | 5 | | | | 5 | | | 1 | | | | | 12 | | | | 5 | | | | | | | 4.4 | | | | | | | 0.08 | | | | | |  |  |  |  |
| April | | 0 | | | 3 | | | 0 | | | | 1 | | | 1 | | | | | 6 | | | | no data | | | | | | | 2.0 | | | | | | | 0.034 | | | | | |  |  |  |  |
| May | | 0 | | | 0 | | | 0 | | | | 2 | | | 0 | | | | | 17 | | | | no data | | | | | | | 3.2 | | | | | | | 0.05 | | | | | |  |  |  |  |
| June | | 0 | | | 0 | | | 0 | | | | 0 | | | 0 | | | | | 20 | | | | no data | | | | | | | 3.3 | | | | | | | 0.06 | | | | | |  |  |  |  |
| July | | 0 | | | 1 | | | 0 | | | | 0 | | | 0 | | | | | 16 | | | | no data | | | | | | | 2.9 | | | | | | | 0.05 | | | | | |  |  |  |  |
| August | | 1 | | | 1 | | | 2 | | | | 1 | | | 1 | | | | | 3 | | | | no data | | | | | | | 1.5 | | | | | | | 0.027 | | | | | |  |  |  |  |
|  | | 29 | | | 53 | | | 40 | | | | 60 | | | 36 | | | | | 116 | | | | 32 | | | | | | | 57.9 | | | | | | | 1 | | | | | |  |  |  |  |
|  | | Total outbreaks 365 | | | | | | | | | |  | | | | | | | | | | | |  | | | | | | |  | | | | | | |  | | | | | |  |  |  |  |

| **Hungary 2005** | Median monthly outbreak percentage: 0.072993 | | | | | | |  |
| --- | --- | --- | --- | --- | --- | --- | --- | --- |
| Month | 1998 | 1999 | 2000 | 2001 | 2002 | 2003 | Total | %/month |
| September | 0 | 0 | 1 | 5 | 6 | 3 | 15 | 0.05 |
| October | 0 | 1 | 2 | 6 | 8 | 3 | 20 | **0.072993 SMSP** |
| November | 2 | 0 | 1 | 3 | 16 | 11 | 33 | 0.12 |
| December | 1 | 0 | 1 | 5 | 16 | 2 | 25 | 0.09 |
| January | 0 | 1 | 0 | 0 | 19 | 27 | 47 | **0.17 POM** |
| February | 0 | 0 | 0 | 2 | 19 | 19 | 40 | 0.15 |
| March | 0 | 0 | 1 | 3 | 15 | 12 | 31 | 0.11 |
| April | 0 | 0 | 0 | 4 | 9 | 4 | 17 | 0.06 |
| May | 0 | 1 | 4 | 8 | 4 | 3 | 20 | 0.07 |
| June | 0 | 1 | 0 | 6 | 3 | 1 | 11 | 0.04 |
| July | 0 | 1 | 1 | 4 | 2 | 2 | 10 | 0.04 |
| August | 0 | 1 | 0 | 1 | 2 | 1 | 5 | 0.02 |
|  | 3 | 6 | 11 | 47 | 119 | 88 | 274 | 1 |

| **Hungary 2009** | Median monthly outbreak percentage: 0.06 | | | | | |  |  | |  | |  |
| --- | --- | --- | --- | --- | --- | --- | --- | --- | --- | --- | --- | --- |
| Month | 2001 | 2002 | 2003 | 2004 | 2005 | 2006 | 2007 | | Avg/month | | %/month | |
| September | 5 | 5 | 3 | 3 | 0 | 8 | no data | | 4.0 | | 0.04 | |
| October | 5 | 5 | 2 | 5 | 0 | 10 | no data | | 4.5 | | 0.04 | |
| November | 4 | 17 | 11 | 10 | 3 | 48 | no data | | 15.5 | | **0.14 SMSP** | |
| December | 6 | 20 | 2 | 17 | 10 | 39 | no data | | 15.7 | | 0.15 | |
| January | 0 | 19 | 23 | 17 | 14 | 18 | 50 | | 20.1 | | **0.19 POM** | |
| February | 2 | 18 | 20 | 6 | 14 | 13 | 30 | | 14.7 | | 0.14 | |
| March | 3 | 13 | 11 | 11 | 4 | 14 | 23 | | 11.3 | | 0.10 | |
| April | 4 | 10 | 3 | 4 | 5 | 7 | 23 | | 8.0 | | 0.07 | |
| May | 9 | 3 | 2 | 8 | 2 | 3 | no data | | 4.5 | | 0.04 | |
| June | 6 | 3 | 1 | 1 | 0 | 11 | no data | | 3.7 | | 0.03 | |
| July | 4 | 2 | 2 | 1 | 0 | 11 | no data | | 3.3 | | 0.03 | |
| August | 0 | 2 | 1 | 4 | 1 | 7 | no data | | 2.5 | | 0.02 | |
|  | 48 | 117 | 81 | 87 | 53 | 189 | 126 | | 108 | | 1 | |
|  | Total outbreaks 701 | | | | | |  | |  | |  | |

| **Japan, Osaka City 2002** | | Median monthly outbreak percentage: 0.02 | | | | |  |  |
| --- | --- | --- | --- | --- | --- | --- | --- | --- |
| Month | 1996 | 1997 | 1998 | | 1999 | 2000 | Avg/month | %/month |
| September | 0 | 0 | 0 | | 0 | no data | 0 | 0.000 |
| October | 0 | 0 | 0 | | 0 | no data | 0 | 0.000 |
| November | 0 | 0 | 2 | | 0 | no data | 1 | **0.03 SMSP** |
| December | 0 | 2 | 1 | | 5 | no data | 2 | 0.12 |
| January | no data | 5 | 10 | | 3 | 10 | 7 | **0.43 POM** |
| February | no data | 10 | 1 | | 1 | 2 | 4 | 0.22 |
| March | no data | 3 | 5 | | 1 | 1 | 3 | 0.15 |
| April | 0 | 1 | 0 | | 0 | no data | 0 | 0.02 |
| May | 0 | 0 | 0 | | 0 | no data | 0 | 0.000 |
| June | 1 | 0 | 0 | | 0 | no data | 0 | 0.02 |
| July | 1 | 0 | 0 | | 0 | no data | 0 | 0.02 |
| August | 0 | 0 | 0 | | 0 | no data | 0 | 0.000 |
|  | 2 | 21 | 19 | | 10 | 13 | 16 | 1.0 |
|  | Total outbreaks 65 | | |  | |  |  |  |

|  | |  | |  | |  | |  | |  | |  | |  | |  | |  |
| --- | --- | --- | --- | --- | --- | --- | --- | --- | --- | --- | --- | --- | --- | --- | --- | --- | --- | --- |
|  | | | | | | | | | |  | |  | |  | |  | |  |
| **Japan, Osaka City 2009** | | | | Median monthly outbreak percentage 0.05 | | | | | | | | | |  | |  | |  |
| Month | 2001 | | 2002 | | 2003 | | 2004 | | 2005 | | 2006 | | 2007 | | Avg/month | | %/month | |
| September | 0 | | 1 | | 1 | | 0 | | 1 | | 2 | | no data | | 0.8 | | 0.02 | |
| October | 1 | | 0 | | 0 | | 0 | | 2 | | 6 | | no data | | 1.5 | | 0.03 | |
| November | 3 | | 1 | | 2 | | 1 | | 4 | | 34 | | no data | | 7.5 | | **0.16 SMSP** | |
| December | 7 | | 2 | | 10 | | 12 | | 23 | | 7 | | no data | | 10.2 | | 0.21 | |
| January | 10 | | 9 | | 7 | | 6 | | 18 | | 15 | | 11 | | 10.9 | | **0.23 POM** | |
| February | 5 | | 7 | | 12 | | 6 | | 10 | | 3 | | 3 | | 6.6 | | 0.14 | |
| March | 2 | | 2 | | 7 | | 5 | | 4 | | 5 | | 4 | | 4.1 | | 0.09 | |
| April | 0 | | 0 | | 1 | | 5 | | 3 | | 5 | | no data | | 2.3 | | 0.05 | |
| May | 0 | | 0 | | 0 | | 6 | | 3 | | 7 | | no data | | 2.7 | | 0.06 | |
| June | 0 | | 0 | | 0 | | 0 | | 1 | | 5 | | no data | | 1.0 | | 0.02 | |
| July | 0 | | 0 | | 1 | | 0 | | 0 | | 0 | | no data | | 0.2 | | 0.003 | |
| August | 0 | | 0 | | 0 | | 0 | | 0 | | 0 | | no data | | 0.0 | | 0.000 | |
|  | 28 | | 22 | | 41 | | 41 | | 69 | | 89 | | 18 | | 48 | | 1 | |
|  | Total outbreaks 308 | | | | | |  | |  | | | |  | |  | | | |

| **Netherlands** | | Median monthly outbreak percentage 0.063 | | | | | | |  |  |  |  |  |  |
| --- | --- | --- | --- | --- | --- | --- | --- | --- | --- | --- | --- | --- | --- | --- |
| Month | 1994 | 1995 | 1996 | 1997 | 1998 | 1999 | 2000 | 2001 | 2002 | 2003 | 2004 | 2005 | Total | %/month |
| September | 1 | 1 | 0 | 0 | 0 | 1 | 2 | 1 | 5 | 1 | 9 | 0 | 21 | 0.03 |
| October | 0 | 0 | 1 | 4 | 0 | 2 | 1 | 4 | 10 | 1 | 19 | 1 | 43 | **0.064 SMSP** |
| November | 1 | 6 | 2 | 2 | 2 | 3 | 4 | 7 | 35 | 4 | 33 | 2 | 101 | 0.15 |
| December | 0 | 9 | 3 | 3 | 2 | 9 | 8 | 9 | 59 | 3 | 34 | 11 | 150 | **0.22 POM** |
| January | 3 | 3 | 18 | 2 | 2 | 3 | 0 | 6 | 20 | 18 | 8 | 32 | 115 | 0.17 |
| February | 1 | 3 | 17 | 1 | 0 | 3 | 6 | 8 | 10 | 11 | 4 | 12 | 76 | 0.11 |
| March | 1 | 3 | 10 | 4 | 3 | 6 | 8 | 6 | 6 | 1 | 3 | 8 | 59 | 0.09 |
| April | 2 | 0 | 1 | 1 | 0 | 2 | 5 | 4 | 13 | 4 | 2 | 7 | 41 | 0.06 |
| May | 0 | 2 | 3 | 1 | 0 | 0 | 3 | 1 | 8 | 1 | 3 | 2 | 24 | 0.04 |
| June | 0 | 1 | 1 | 0 | 1 | 0 | 0 | 2 | 3 | 0 | 4 | 1 | 13 | 0.02 |
| July | 0 | 0 | 0 | 2 | 1 | 0 | 1 | 0 | 3 | 0 | 2 | 3 | 12 | 0.02 |
| August | 0 | 1 | 0 | 0 | 1 | 3 | 1 | 1 | 3 | 0 | 3 | 2 | 15 | 0.02 |
|  | 9 | 29 | 56 | 20 | 12 | 32 | 39 | 49 | 175 | 44 | 124 | 81 | 670 | 1 |

| **New Zealand** |  | Median monthly outbreak percentage 0.0825 | | | | |  |  |  |  |  |
| --- | --- | --- | --- | --- | --- | --- | --- | --- | --- | --- | --- |
| Month | 2002 | 1997 | 1998 | 2005 | 2006 | 2007 | 2008 | 2009 | Total | %/month | |
| February | 2 | 3 | 3 | 2 | 15 | 14 | 8 | 2 | 49 | 0.04 | |
| March | 9 | 4 | 9 | 8 | 27 | 11 | 11 | 17 | 96 | 0.0796 | |
| April | 6 | 8 | 20 | 2 | 20 | 10 | 15 | 17 | 98 | 0.08 | |
| May | 6 | 10 | 13 | 4 | 14 | 11 | 15 | 22 | 95 | 0.0788 | |
| June | 10 | 12 | 28 | 5 | 11 | 9 | 10 | 25 | 110 | **0.09 SMSP** | |
| July | 5 | 7 | 21 | 3 | 7 | 9 | 16 | 32 | 100 | 0.0829 | |
| August | 5 | 3 | 31 | 1 | 13 | 13 | 15 | 29 | 110 | 0.09 | |
| September | 9 | 4 | 21 | 2 | 15 | 15 | 10 | 26 | 102 | 0.08 | |
| October | 10 | 7 | 20 | 5 | 20 | 32 | 16 | 34 | 144 | **0.12 POM** | |
| November | 10 | 4 | 10 | 8 | 14 | 26 | 20 | 33 | 125 | 0.10 | |
| December | 8 | 6 | 14 | 7 | 17 | 20 | 7 | 20 | 99 | 0.08 | |
| January | 1 | 8 | 5 | 11 | 7 | 23 | 16 | 7 | 78 | 0.06 | |
|  | 81 | 76 | 195 | 58 | 180 | 193 | 159 | 264 | 1206 | 1 | |

|  |  |  |  |  |  |  |  |  |
| --- | --- | --- | --- | --- | --- | --- | --- | --- |
| **Norway** | Median monthly outbreak percentage 0.05 | | | |  |  |  | |
| Month | 2001 | 2002 | 2003 | 2004 | 2005 | Avg/month | %/month | |
| September | 0 | 5 | 0 | 1 | no data | 1.5 | 0.03 | |
| October | 0 | 3 | 2 | 0 | no data | 1.3 | 0.03 | |
| November | 0 | 12 | 2 | 4 | no data | 4.5 | **0.10 SMSP** | |
| December | 1 | 34 | 6 | 8 | no data | 12.3 | **0.28 POM** | |
| January | 4 | 1 | 21 | 0 | 6 | 6.4 | 0.15 | |
| February | 2 | 1 | 13 | 1 | 5 | 4.4 | 0.10 | |
| March | 7 | 2 | 8 | 4 | 4 | 5.0 | 0.12 | |
| April | 4 | 3 | 3 | 1 | 1 | 2.4 | 0.06 | |
| May | 3 | 2 | 1 | 0 | 2 | 1.6 | 0.04 | |
| June | 0 | 0 | 2 | 1 | 0 | 0.6 | 0.01 | |
| July | 2 | 2 | 3 | 1 | 0 | 1.6 | 0.04 | |
| August | 0 | 2 | 1 | 4 | 1 | 1.6 | 0.04 | |
|  | 23 | 67 | 62 | 25 | 19 | 43.1 | 1 | |
|  | Total outbreaks 196 | | |  |  |  |  | |

| **Spain, Catalonia** | | Median monthly outbreak percentage 0.09 | | | | |  |
| --- | --- | --- | --- | --- | --- | --- | --- |
| Month | 2010 | | 2011 | 2012 | Total | %.month | |
| September | 2 | | 2 | 1 | 5 | 0.04 | |
| October | 2 | | 3 | 9 | 14 | 0.11 | |
| November | 6 | | 6 | 5 | 17 | **0.13 POM** | |
| December | 5 | | 1 | 1 | 7 | 0.05 | |
| January | 10 | | 3 | 1 | 14 | **0.11 SMSP** | |
| February | 8 | | 5 | 1 | 14 | 0.11 | |
| March | 12 | | 2 | 1 | 15 | 0.12 | |
| April | 5 | | 1 | 2 | 8 | 0.06 | |
| May | 5 | | 1 | 5 | 11 | 0.09 | |
| June | 4 | | 4 | 3 | 11 | 0.09 | |
| July | 4 | | 5 | 0 | 9 | 0.07 | |
| August | 0 | | 1 | 2 | 3 | 0.02 | |
|  | 63 | | 34 | 31 | 128 | 1 | |

| **Spain, Catalonia and Valencia,** | | | Median monthly outbreak percentage 0.08 | | | | |  |
| --- | --- | --- | --- | --- | --- | --- | --- | --- |
| Month | 2001 | 2002 | 2003 | 2004 | 2005 | 2006 | Avg/month | %/month |
| September | 2 | 2 | 1 | 0 | 6 | 3 | 2.3 | 0.07 |
| October | 0 | 3 | 3 | 6 | 5 | 6 | 3.8 | 0.11 |
| November | 1 | 2 | 4 | 8 | 1 | 5 | 3.5 | 0.10 |
| December | 0 | 0 | 0 | 4 | 6 | 4 | 2.3 | 0.07 |
| January | no data | 1 | 5 | 2 | 1 | 11 | 4.0 | 0.12 |
| February | no data | 1 | 5 | 0 | 8 | 8 | 4.4 | **0.13 POM** |
| March | 2 | 1 | 0 | 0 | 2 | 5 | 1.7 | 0.049 |
| April | 0 | 2 | 2 | 1 | 3 | 3 | 1.8 | 0.05 |
| May | 5 | 5 | 0 | 0 | 4 | 4 | 3.0 | 0.09 |
| June | 1 | 1 | 0 | 0 | 5 | 3 | 1.7 | 0.049 |
| July | 1 | 5 | 1 | 1 | 6 | 0 | 2.3 | 0.07 |
| August | 1 | 3 | 1 | 2 | 6 | 4 | 2.8 | 0.08 |
|  | 13 | 26 | 22 | 24 | 53 | 56 | 33.7 | 1 |
|  | Total outbreaks 194 (chart in study had 195 norovirus outbreaks, though study listed total outbreaks as 169) | | | | | | | |

| **US 2002** | Median monthly outbreak percentage 0.082 | | | |  |  |
| --- | --- | --- | --- | --- | --- | --- |
| Month | 1997 | 1998 | 1999 | 2000 | Avg/month | %/month |
| September | 4 | 6 | 3 | no data | 4 | 0.07 |
| October | 5 | 4 | 4 | no data | 4 | 0.07 |
| November | 8 | 9 | 2 | no data | 6 | 0.10 |
| December | 4 | 5 | 14 | no data | 8 | **0.12 POM** |
| January | no data | 1 | 6 | 5 | 4 | 0.06 |
| February | no data | 1 | 9 | 11 | 7 | **0.11 SMSP** |
| March | no data | 6 | 5 | 9 | 7 | 0.10 |
| April | no data | 4 | 6 | 8 | 6 | 0.09 |
| May | no data | 3 | 9 | 4 | 5 | 0.08 |
| June | no data | 4 | 7 | no data | 6 | 0.08 |
| July | 6 | 2 | 4 | no data | 4 | 0.06 |
| August | 2 | 6 | 7 | no data | 5 | 0.08 |
|  | 29 | 51 | 76 | 37 | 66 | 1 |
|  | Total outbreaks 193 | | |  |  |  |

| **US 2006** | | Median monthly outbreak percentage 0.078 | | | | | | | |  | |  | | | |  | |  |
| --- | --- | --- | --- | --- | --- | --- | --- | --- | --- | --- | --- | --- | --- | --- | --- | --- | --- | --- |
| Month | | 2000 | | 2001 | | 2002 | | 2003 | | 2004 | | Avg/month | | | | %/month | | |
| September | | 4 | | 1 | | 2 | | 2 | | no data | | 2 | | | | 0.05 | | |
| October | | 1 | | 2 | | 8 | | 3 | | no data | | 4 | | | | 0.07527 | | |
| November | | 3 | | 2 | | 8 | | 2 | | no data | | 4 | | | | **0.0806 SMSP** | | |
| December | | 12 | | 2 | | 7 | | 3 | | no data | | 6 | | | | 0.13 | | |
| January | | no data | | 7 | | 0 | | 14 | | 6 | | 7 | | | | 0.15 | | |
| February | | no data | | 5 | | 3 | | 3 | | 18 | | 7 | | | | **0.16 POM** | | |
| March | | no data | | 6 | | 4 | | 4 | | 6 | | 5 | | | | 0.11 | | |
| April | | no data | | 4 | | 5 | | 2 | | 4 | | 4 | | | | 0.08 | | |
| May | | no data | | 3 | | 1 | | 3 | | 3 | | 3 | | | | 0.05 | | |
| June | | no data | | 3 | | 1 | | 2 | | 1 | | 2 | | | | 0.04 | | |
| July | | 4 | | 0 | | 4 | | 1 | | no data | | 2.250 | | | | 0.05 | | |
| August | | 4 | | 1 | | 1 | | 1 | | no data | | 1.750 | | | | 0.04 | | |
|  | | 28 | | 36 | | 44 | | 40 | | 38 | | 47 | | | | 1 | | |
|  | | Total outbreaks 186 | | | | | |  | |  | |  | | | |  | | |
| **US 2011** | Median monthly outbreak percentage 0.05 | | | | | | | | | |  | | |  |  |  |  |  |
| Month | | 2007 | 2008 | | 2009 | | 2010 | | Avg/month | | | | %/month | | | |  |  |
| September | | 20 | 29 | | 12.5 | | no data | | 20 | | | | 0.03 | | | |  |  |
| October | | 15 | 58 | | 17.5 | | no data | | 30 | | | | 0.04 | | | |  |  |
| November | | 30 | 70 | | 52.5 | | no data | | 51 | | | | **0.06 SMSP** | | | |  |  |
| December | | 80 | 148 | | 117.5 | | no data | | 115 | | | | 0.14 | | | |  |  |
| January | | 215 | 145 | | 200 | | 118 | | 169 | | | | **0.21 POM** | | | |  |  |
| February | | 183 | 103 | | 155 | | 163 | | 151 | | | | 0.19 | | | |  |  |
| March | | 108 | 100 | | 107.5 | | 125 | | 110 | | | | 0.14 | | | |  |  |
| April | | 63 | 95 | | 55 | | 53 | | 66 | | | | 0.08 | | | |  |  |
| May | | 40 | 45 | | 15 | | no data | | 33 | | | | 0.04 | | | |  |  |
| June | | 23 | 25 | | 7.5 | | no data | | 18.33 | | | | 0.02 | | | |  |  |
| July | | 10 | 28 | | 17.5 | | no data | | 18.33 | | | | 0.02 | | | |  |  |
| August | | 13 | 29 | | 12.5 | | no data | | 17.92 | | | | 0.02 | |  |  |  |  |
|  | | 798 | 873 | | 770 | | 458 | | 800 | | | | 1 | |  |  |  |  |
|  | | Total outbreaks 2898 | | | | |  | |  | | | |  | |  |  |  |  |

**Appendix 4. Monthly number and monthly percentage of norovirus outbreak by setting, Alberta, Canada, 2002 – 2012.**

| **Month** | **Health Institution** | | **Child Care** | | **Schools** | | **Food**  **Facility** | | **Community** | | **Unknown^a^** | | **Other^b^** | | **Total** |
| --- | --- | --- | --- | --- | --- | --- | --- | --- | --- | --- | --- | --- | --- | --- | --- |
| Sep | 27 | 2% | **4** | **10%** | **3** | **7%** | 0 | 0% | 2 | 9% | 0 | 0% | 1 | 3% | 37 |
| Oct | 73 | 5% | **13** | **32%** | **8** | **20%** | 6 | 13% | 4 | 17% | 0 | 0% | 1 | 3% | 105 |
| Nov | **271** | **18%** | **8** | **20%** | **11** | **27%** | 2 | 4% | 1 | 4% | 0 | 0% | 3 | 8% | 296 |
| Dec | **359** | **24%** | 0 | 0% | **9** | **22%** | 5 | 11% | 2 | 9% | 4 | 33% | 7 | 18% | 386 |
| Jan | **286** | **20%** | 2 | 5% | 0 | 0% | 0 | 0% | 2 | 9% | 3 | 25% | 3 | 8% | 296 |
| Feb | **124** | **8%** | 4 | 10% | 3 | 7% | 5 | 11% | 0 | 0% | 0 | 0% | 0 | 0% | 136 |
| Mar | **109** | **7%** | 0 | 0% | 2 | 5% | 5 | 11% | 4 | 17% | 1 | 8% | 3 | 8% | 124 |
| April | 67 | 5% | 1 | 2% | 2 | 5% | 6 | 13% | 1 | 4% | 0 | 0% | 3 | 8% | 80 |
| May | 59 | 4% | 4 | 10% | 1 | 2% | 5 | 11% | 2 | 9% | 2 | 17% | 4 | 10% | 77 |
| Jun | 33 | 2% | 3 | 7% | 2 | 5% | 8 | 17% | 2 | 9% | 1 | 8% | 3 | 8% | 52 |
| Jul | 36 | 2% | 1 | 2% | 0 | 0% | 2 | 4% | 2 | 9% | 0 | 0% | 4 | 10% | 45 |
| Aug | 22 | 2% | 1 | 2% | 0 | 0% | 3 | 6% | 1 | 4% | 1 | 8% | 7 | 18% | 35 |
| Total | 1466 | 100% | 41 | 100% | 41 | 100% | 47 | 100% | 23 | 100% | 12 | 100% | 39 | 100 100% | 1669 |
| median | 5% | | 6% | | 5% | | 11% | | 9% | | 4% | | 8% | |  |

Bold indicates months of seasonal peak

^a^ outbreaks with unknown settings

| ^b^ Group home (7), shelter (7), camp (6), hotel (6), restricted function (5), travel (4), correction facility (2), treatment centre (2) |
| --- |
